# Theoretical study on the polar hydrogen-π (Hp-π) interactions between protein side chains

**DOI:** 10.1186/1752-153X-7-92

**Published:** 2013-05-25

**Authors:** Qi-Shi Du, Qing-Yan Wang, Li-Qin Du, Dong Chen, Ri-Bo Huang

**Affiliations:** 1 State Key Laboratory of Non-food Biomass and Enzyme Technology, National Engineering Research Center for Non-food Biorefinery, Guangxi Academy of Sciences, 98 Daling Road, Nanning, Guangxi 530007, China; 2 Life Science and Biotechnology College, Guangxi University, Nanning, Guangxi, 530004, China; 3 Gordon Life Science Institute, San Diego, CA 92130, USA

**Keywords:** Protein structures, Molecular interactions, Hydrogen-π interactions, Protein backbones, CCSD, Ghost atom

## Abstract

**Background:**

In the study of biomolecular structures and interactions the polar hydrogen-π bonds (Hp-π) are an extensive molecular interaction type. In proteins 11 of 20 natural amino acids and in DNA (or RNA) all four nucleic acids are involved in this type interaction.

**Results:**

The Hp-π in proteins are studied using high level QM method CCSD/6-311 + G(d,p) + H-Bq (ghost hydrogen basis functions) in vacuum and in solutions (water, acetonitrile, and cyclohexane). Three quantum chemical methods (B3LYP, CCSD, and CCSD(T)) and three basis sets (6-311 + G(d,p), TZVP, and cc-pVTZ) are compared. The Hp-π donors include R_2_NH, RNH_2_, ROH, and C_6_H_5_OH; and the acceptors are aromatic amino acids, peptide bond unit, and small conjugate π-groups. The Hp-π interaction energies of four amino acid pairs (Ser-Phe, Lys-Phe, His-Phe, and Tyr-Phe) are quantitatively calculated.

**Conclusions:**

Five conclusion points are abstracted from the calculation results. (1) The common DFT method B3LYP fails in describing the Hp-π interactions. On the other hand, CCSD/6-311 + G(d,p) plus ghost atom H-Bq can yield better results, very close to the state-of-the-art method CCSD(T)/cc-pVTZ. (2) The Hp-π interactions are point to π-plane interactions, possessing much more interaction conformations and broader energy range than other interaction types, such as common hydrogen bond and electrostatic interactions. (3) In proteins the Hp-π interaction energies are in the range 10 to 30 kJ/mol, comparable or even larger than common hydrogen bond interactions. (4) The bond length of Hp-π interactions are in the region from 2.30 to 3.00 Å at the perpendicular direction to the π-plane, much longer than the common hydrogen bonds (~1.9 Å). (5) Like common hydrogen bond interactions, the Hp-π interactions are less affected by solvation effects.

## Background

The structures of proteins and other biological molecules are determined by the delicate balance between several molecular interactions [1-3]. Among them hydrogen-π interactions [4-7] are an extensive interaction type in organic and biological molecules, referring to the interactions between hydrogen atoms, attaching to different atomic groups, and aromatic molecules or π-groups. The hydrogen-π interactions can be classified into two groups, the nonpolar hydrogen-π interactions (H-π or CH-π) [8-11] and the polar hydrogen-π interactions (Hp-π) [12-16]. The typical nonpolar hydrogen-π interactions are the interactions between hydrogen atoms, attaching to carbon atoms, and the conjugate π-systems, often indicated by the notation CH-π in references [8,11]. The interaction strength and the physical nature and properties of nonpolar H-π interactions are often a debatable topic [6,10,17]. In the polar hydrogen-π interactions the donors are the polar hydrogen atoms, attaching to electronegative atoms (R_2_NH, RNH_2_, and ROH), and the acceptors are various aromatic molecules and conjugate π-groups [18-20]. The interaction energies of polar hydrogen-π interactions are much stronger than that of nonpolar hydrogen-π interactions (CH-π), comparable or even larger than common hydrogen bonds. In this study the notation Hp-π is used for the polar hydrogen-π interactions, to make difference from the nonpolar hydrogen-π interactions (H-π or CH-π), and the common hydrogen bond interactions (H-b) [21-25].

The Hp-π interactions frequently happen in biological macromolecules, such as proteins and DNA (or RNA), and play important roles in protein structures and biological functions. In proteins the Hp-π interaction donors are the polar hydrogen atoms in atomic groups [12-16] –NH_2_, >NH, –OH, –SH, and C_6_H_5_OH, which exist in amino acids Ser, Thr, Tyr, Trp, Cys, His, Asn, Gln, Lys, and Arg, while the acceptors are the aromatic amino acids, Phe, Tyr, Trp, and His [19,20]. Three amino acids play the roles of both donor and acceptor (His, Tyr, and Trp). In the 20 natural amino acids 11 of them may be involved in the Hp-π interactions. The peptide bond units are a quasi π-group, comprising N, C, and O, which can play the roles of both Hp-π interaction acceptor and donor [6]. In DAN and RNA the four nucleic acid components (adenine, guanine, cytosine, and thymine) possess aromatic rings and polar hydrogen groups, which are the Hp-π interaction acceptors and donors [23].

The Hp-π interactions are a unique interaction type that cannot be classified into other molecular interaction types, such as common hydrogen bond [21-25], cation-π interaction [26-29], electrostatic interaction, and van der Waals interaction. The physical nature and the interaction strength of Hp-π interactions are often a debatable topic. In the Hp-π interactions electron dispersion energy is one of the main contributions. Different quantum chemical methods may give very different interaction energies and descriptions for the interaction properties of Hp-π interactions. One reason is that some quantum chemical methods and basis sets may fail in estimating the dispersion energies accurately [30]. In this study several quantum chemical methods and basis sets are evaluated and compared in the Hp-π interaction calculations. The Hp-π interaction contributors, investigated in this study, include the interaction donors R_2_NH, RNH_2_, ROH, C_6_H_5_OH, and peptide bond unit NMA (*n*-methyl acetamide); and the interaction acceptors include aromatic molecular benzene, hetero-aromatic molecules C_5_H_5_N and imidazole, peptide bond unit, carbonyl group, and other small conjugate π-groups.

## Results

In this section all calculation results are summarized and reported using tables and figures. Brief comparisons and illustrations are provided following the results.

### Hp-π interactions of small conjugate π-groups

**Figure 1:**
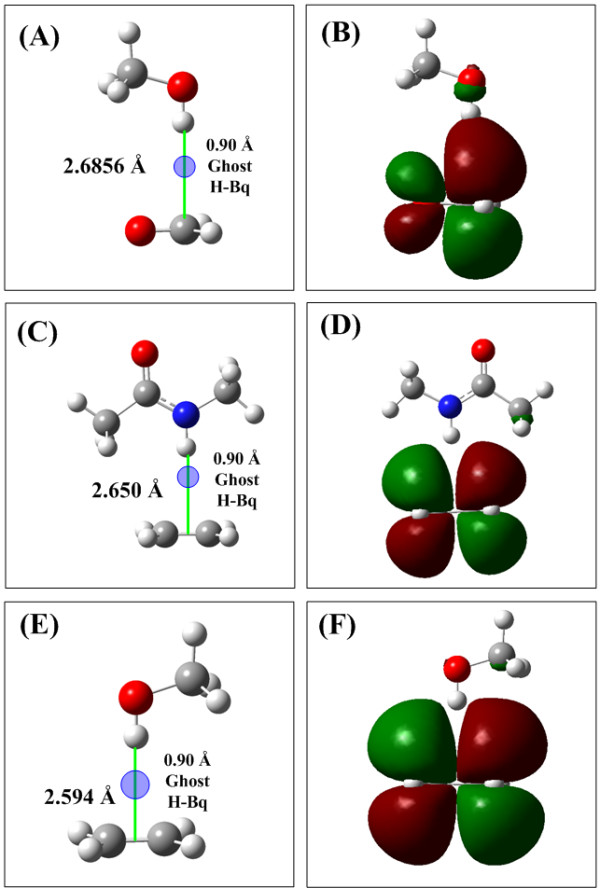
**The Hp-π interaction structures and HOMOs of three small interaction pairs.** (**A**) Structure of Hp-π interaction pair CH_3_OH-H_2_CO. The polar hydrogen atom of CH_3_OH points to the carbon atom of H_2_CO perpendicularly. (**B**) The HOMO of Hp-π interaction pair CH_3_OH-H_2_CO. The polar hydrogen atom is in close touching with the π-orbital of H_2_CO. (**C**) Structure of Hp-π interaction pair NMA-C_2_H_4_. The polar hydrogen atom of NMA (*n*-methyl acetamide) points to the center of double bond. (**D**) The HOMO of Hp-π interaction pair NMA-C_2_H_4_. (**E**) Structure of Hp-π interaction pair CH_3_OH-C_2_H_4_. The polar hydrogen atom of CH_3_OH points to the center of double bond perpendicularly. (**F**) The HOMO of Hp-π interaction pair CH_3_OH-C_2_H_4_. The ghost hydrogen atom H-Bq is attached to the polar hydrogen atoms, and the distance to polar hydrogen is 0.90 Å.

In this section the Hp-π interactions between small donors and acceptors are used to illustrate the Hp-π interaction properties and strength, including three Hp-π interaction pairs CH_3_OH–H_2_CO, NMA–C_2_H_4_, and CH_3_OH–C_2_H_4_. The Hp-π interaction structures of three Hp-π interaction pairs are shown in Figure 1, and the Hp-π interaction energies are listed in Table 1. In Figure 1 the small blue circle are ‘ghost’ hydrogen atoms, which improve the Hp-π QM calculations effectively. The role of ‘ghost’ atoms will be introduced in detail in the Method section. The HOMOs (highest occupied molecular orbitals) of three Hp-π interaction pairs are shown in Figure 1, in which the polar hydrogen atoms are in close touching with the π-orbitals of the aromatic molecules. In other words, in the Hp-π interactions the polar hydrogen atoms are buried in the π-electron density of aromatic molecules, similar to the common hydrogen bond interactions.

**Table 1:** **The polar hydrogen-π (Hp-π) interactions between three small molecular interaction pairs (CH**_**3**_**OH–H**_**2**_**CO, NMA–C**_**2**_**H**_**4**_**, and CH**_**3**_**OH–C**_**2**_**H**_**4**_**)**

**Vacuum**	**CH**_**3**_**OH–H**_**2**_**CO**		**NMA–C**_**2**_**H**_**4**_		**CH**_**3**_**OH–C**_**2**_**H**_**4**_	
	**C**	**O**	**C**	**Bond**	**C**	**Bond**
^a^ Energy	-3.6370	-11.0838	-11.2956	-12.8848	-11.3071	-12.8871
^b^ Bond	2.6856	2.3277	2.6500	2. 5943	2.6453	2.5354
^**c **^**Solvent**	**Acting position**	**Water (ϵ = 78.39)**	**Acetonitrile (ϵ = 35.9)**		**Cyclohexane (ϵ = 2.0)**	
CH_3_OH-CH_2_O	Oxygen	-2.3002	-2.5012		-6.7355	
CH_3_OH-C_2_H_4_	Bond center	-6.8966	-6.9797		-8.8056	

^a ^Interaction energies are in kJ/mol, calculated using CCSD/6-311 + G(d,p) plus ghost atom H-Bq.

^b ^Bond lengths are in angstrom (Å).

^c ^Energies in solutions are calculated using CCSD + PCM method.

Unlike common hydrogen bond interactions that are point-to-point interactions, the Hp-π interactions are point to π-group interactions, in which the interactions could happen at any position of the π-plane. In Table 1 for each Hp-π interaction pair two or more interaction positions are reported. In the NMA–C_2_H_4_ interaction pair the Hp-π energy at the bond center (-12.885 kJ/mol) is higher than that (-11.296 kJ/mol) at the carbon atom of C_2_H_4_. The structural conformations of Hp-π interactions are much more than that of the common hydrogen bond, and the Hp-π interaction energies could change in a broad range. The Hp-π interaction energies of all three small interaction pairs are larger than half of H_2_O–H_2_O hydrogen bond. The NMA (*n*-methyl acetamide) [31-33] is used as the model of peptide bond units. The Hp-π energy of NMA may represent the Hp-π interactions between the protein peptide backbones and aromatic side chains. The lower part of Table 1 is the Hp-π interactions in three solutions (water, acetonitrile, and cyclohexane) using CCSD and PCM method [34-37]. The Hp-π energies in solutions are decrease with the increase of the solvent dielectric constants ϵ. However, the Hp-π energies in aqueous solution are still large. At this point the Hp-π interactions are like common hydrogen bond interaction, lees affected by solvation effects.

**Figure 2:**
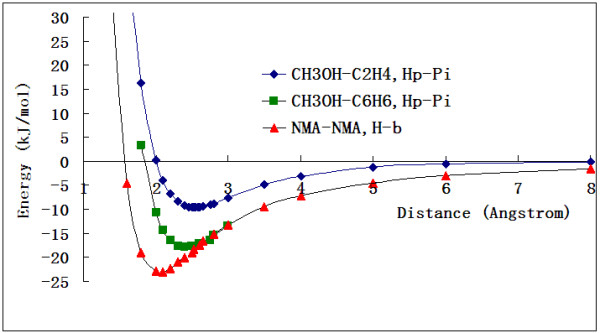
**The Hp-π interaction energies of CH**_**3**_**OH-C**_**2**_**H**_**4 **_**(blue diamond) and CH**_**3**_**OH-C**_**6**_**H**_**6 **_**(green square) pairs as the function of interaction distance (R). **For comparison the curve of NMA-NMA hydrogen bond interaction is also shown (orange triangles), which is the most frequent hydrogen bond in proteins. The bong lengths (~2.5 Å) of Hp-π interactions are longer than that of the hydrogen bonds (~2.0 Å). The force constants (k) of two Hp-π interaction pairs CH_3_OH-C_2_H_4 _and CH_3_OH-C_6_H_6 _are 0.0035 and 0.0055 Hartree/Bohr, smaller that (0.0071 Hartree/Bohr) of the NMA-NMA hydrogen bond interaction. The Hp-π interactions are more soften than the common hydrogen bond interactions in the minimum and short interaction distances.

The Hp-π interaction energies as the functions of interaction distances (*R*) are shown in Figure 2. For comparison the curve of NMA-NMA hydrogen bond interaction is also shown in Figure 2, which is the most frequent hydrogen bond in proteins. The bong lengths (~2.5 Å) of Hp-π interactions are longer than that of the hydrogen bonds (~2.0 Å). The force constants (*k*) of two Hp-π interaction pairs CH_3_OH–C_2_H_4_ and CH_3_OH–C_6_H_6_ are 0.0035 and 0.0055 Hartree/Bohr, smaller that (0.0071 Hartree/Bohr) of the NMA-NMA hydrogen bond interaction. The Hp-π interactions are more soften than the common hydrogen bond interactions in the minimum and short interaction distances.

### Hp-π interactions in aromatic molecules

**Figure 3:**
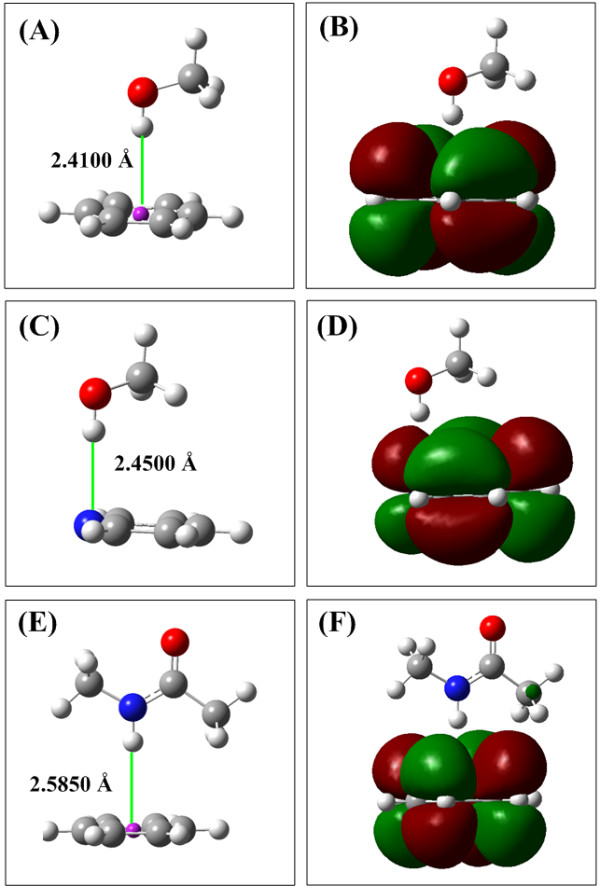
**The Hp-π interaction structures and HOMOs of three aromatic interaction pairs.** (**A**) Structure of Hp-π interaction pair CH_3_OH-C_6_H_6_. The polar hydrogen atom of CH_3_OH points to the center of C_6_H_6 _perpendicularly. (**B**) The HOMO of Hp-π interaction pair CH_3_OH-C_6_H_6_. The polar hydrogen atom is in close touching with the π-orbital of C_6_H_6_. (**C**) Structure of Hp-π interaction pair CH_3_OH-C_5_H_5_N. The polar hydrogen atom of CH_3_OH points to the N of C_5_H_5_N. (**D**) The HOMO of Hp-π interaction pair CH_3_OH-C_5_H_5_N. (**E**) Structure of Hp-π interaction pair NMA-C_6_H_6_. The polar hydrogen atom of NMA (*n*-methyl acetamide) points to the center of C_6_H_6_ perpendicularly. (**F**) The HOMO of Hp-π interaction pair NMA-C_6_H_6_.

The aromatic molecules are the best H-π interaction acceptors. In this section the Hp-π interactions of two aromatic molecules are studied. One is the typical aromatic molecule benzene, and the other is a heteroatom-aromatic molecule C_5_H_5_N. The interaction structures of three interaction pairs (CH_3_OH–C_6_H_6_, CH_3_OH–C_5_H_5_N, and NMA–C_6_H_6_) are shown in Figure 3, and the interaction energies and bond lengths are summarized in Table 2.

**Table 2:** **The interaction energies and bond lengths of three aromatic Hp-π interaction pairs ((CH**_**3**_**)**_**2**_**NH–C**_**6**_**H**_**6**_**, CH**_**3**_**OH–C**_**5**_**H**_**5**_**N, and NMA–C**_**6**_**H**_**6**_**)**

	**(CH**_**3**_**)**_**2**_**NH–C**_**6**_**H**_**6**_	**CH**_**3**_**OH–C**_**5**_**H**_**5**_**N**	**NMA–C**_**6**_**H**_**6**_
Position	Center	N	Center
^a^ Energy	-18.1457	-14.8525	-22.2329
^b^ Bond	2.4100	2.4500	2.5850

^a ^Interaction energies are in kJ/mol, calculated using CCSD/6-311 + G(d,p) plus ghost atom H-Bq.

^b^ Bond lengths are in angstrom (Å).

The H-π interaction energies of benzene are remarkably larger than that of the small Hp-π interaction acceptors (C_2_H_2_ and H_2_CO) listed in Table 1. Usually the H-π interaction energies increase with the size of aromatic molecules. The Hp-π interaction energy (-14.853 kJ/mol) of heteroatom aromatic molecule (C_5_H_5_N) is smaller than the pure aromatic molecule C_6_H_6_ (-22.233 kJ/mol). The Hp-π interaction energy of NMA–C_6_H_6_ (-22.233 kJ/mol) is comparable to the H_2_O-H_2_O hydrogen bond energy (-17 ~ -23 kJ/mol) [38]. This value could represent the Hp-π interaction energies between peptide bond units and the aromatic amino acids in proteins.

### Hp-π interactions in amino acids

**Figure 4:**
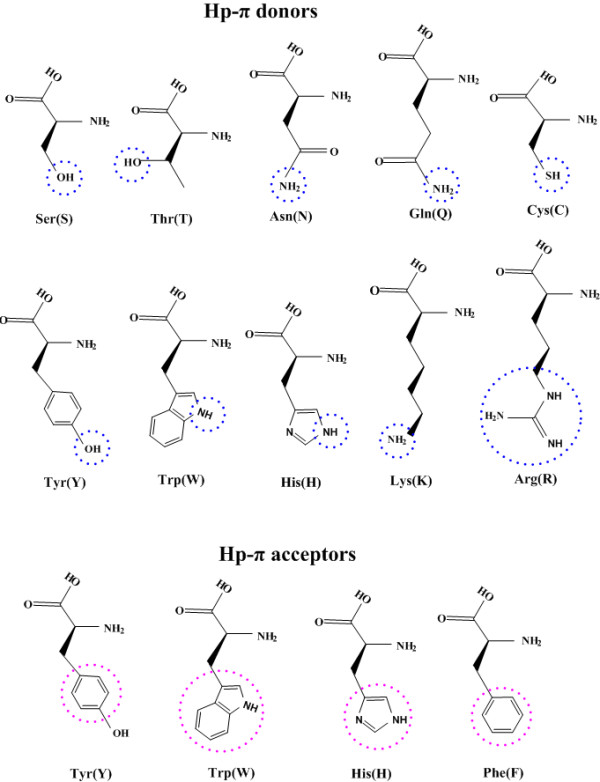
**The Hp-π donors and acceptors of natural amino acids. **In the 20 natural amino acids 4 of them possess aromatic side chains (Phe, Tyr, Trp, and His), which are the possible Hp-π interaction acceptors. On the other hand, 10 amino acids (Ser, Thr, Asn, Gln, Cys, Tyr, Trp, His, Lys, and Arg) possess various Hp-π donors. The atomic group –OH is the Hp-π donor in Ser, Thr, and Tyr. The atomic group > NH or –NH_2 _is the Hp-π donor of Asn, Gln, Lys, Arg, Trp, and His. Amino acid Cys has the donor –SH. Three amino acids (Tyr, Trp, and His) play the roles of both Hp-π donor and acceptor. In proteins total 11 amino acids may be involved in the Hp-π interactions. The Hp-π donors are indicated using blue cycles, and the Hp-π acceptors are indicated using red cycles.

In the 20 natural amino acids 4 of them possess aromatic side chains (Phe, Tyr, Trp, and His), which are the possible Hp-π interaction acceptors. On the other hand, 10 (Ser, Thr, Asn, Gln, Cys, Tyr, Trp, His, Lys, and Arg) amino acids possess various Hp-π donors. The atomic group –OH is the Hp-π donor in amino acids Ser, Thr, and Tyr. The atomic group > NH or –NH_2_ is the Hp-π donor of amino acids Asn, Gln, Lys, Arg, Trp, and His. Amino acid Cys has the Hp-π donor –SH. Three amino acids (Tyr, Trp, and His) play the roles of both Hp-π donor and acceptor. In proteins total 11 amino acids are involved in the Hp-π interactions. The Hp-π interactions of amino acids in proteins are an interesting and important research topic. The amino acid Hp-π donors and acceptors are shown in Figure 4, in which the Hp-π donors are indicated using blue cycles, and the Hp-π acceptors are indicated using red cycles.

**Figure 5:**
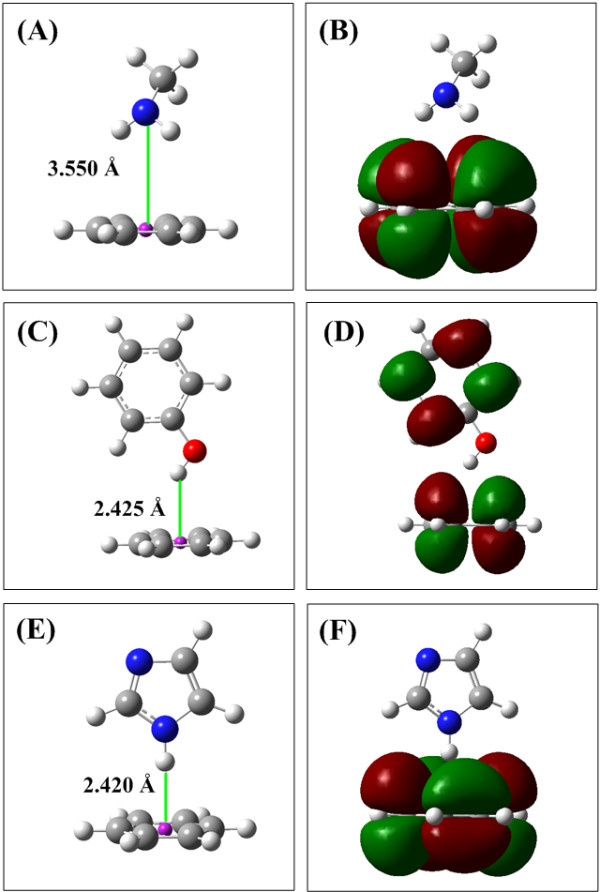
**The Hp-π interaction structures and HOMOs of three amino acid interaction pairs.** (**A**) Structure of Hp-π interaction pair CH_3_NH_2_-C_6_H_6_. The two polar hydrogen atoms of CH_3_NH_2_ point to the benzene ring perpendicularly. (**B**) The HOMO of Hp-π interaction pair CH_3_NH_2_-C_6_H_6_. The polar hydrogen atoms are in close touching with the π-orbital of C_6_H_6_. (**C**) Structure of Hp-π interaction pair C_6_H_5_OH-C_6_H_6_. The polar hydrogen atom of C_6_H_5_OH points to the center of C_6_H_6_. (**D**) The HOMO of Hp-π interaction pair C_6_H_5_OH-C_6_H_6_. (**E**) Structure of Hp-π interaction pair imidazole-C_6_H_6_. The polar hydrogen atom of imidazole points to the center of C_6_H_6 _perpendicularly. (**F**) The HOMO of Hp-π interaction pair imidazole-C_6_H_6_.

In this section the Hp-π interactions of 4 amino acid pairs (Ser–Phe, Lys–Phe, Tyr–Phe, and His–Phe) are studied and reported. The energies and bond lengths of the four amino acid Hp-π interaction pairs are listed in Table 3, and the interaction geometries are shown in Figure 5. In the calculations the aromatic amino acid Phe is simplified as benzene C_6_H_6_, which is the Hp-π acceptor in the four interaction pairs. The four Hp-π donors Ser, Lys, Tyr, and His are simplified as CH_3_OH, CH_3_NH_2_, C_6_H_5_OH, and imidazole, respectively, as shown in Figure 5. Except the Lys–Phe pair, the Hp-π interaction energies of other three amino acid pairs are close or even larger than the H_2_O-H_2_O hydrogen bond energy. In Table 3 the bond length 3.550 Å of Lys–Phe is from N of Lys to the benzene ring center of Phe, and 3.093 Å is the distance from a polar hydrogen atom of Lys to a carbon atom of Phe. The smaller Hp-π interaction energy (-8.766 kJ/mol) of CH_3_NH_2_–C_6_H_6_ pair may indicate that the RNH_2_ is a poorer Hp-π interaction donor than the R_2_NH (-18.15 kJ/mol of (CH_3_)_2_NH–C_6_H_6_).

**Table 3:** The interaction energies and bond lengths of four amino acid Hp-π interaction pairs (Ser–Phe, Lys–Phe, Tyr–Phe, and His–Phe)

	**(Ser–Phe)**	**(Lys–Phe)**	**(Tyr–Phe)**	**(His–Phe)**
	**CH**_**3**_**OH-C**_**6**_**H**_**6**_	**CH**_**3**_**NH**_**2**_**-C**_**6**_**H**_**6**_	**C**_**6**_**H**_**5**_**OH-C**_**6**_**H**_**6**_	**Imid-C**_**6**_**H**_**6**_
Position	Center	Center	Center	Center
^a^ Energy	-19.7648	-8.7661	-21.7831	-25.771
^b^ Bond length	2.410	^c^3.550, 3.093	2.425	2.315

^a ^Interaction energies are in kJ/mol, calculated using CCSD/6-311 + G(d,p) plus ghost atom H-Bq.

^b ^Bond lengths are in angstrom (Å) from polar hydrogen to the center of benzene ring.

^c ^The Bond length 3.550 Å is from N of Lys to the benzene ring center of Phe, and 3.093 Å is the distance from a polar hydrogen atom of Lys to a carbon atom of Phe.

## Discussion

In very recently publications [20] the Hp-π interactions were experimentally and theoretically studied by Kumar and Dasa using resonant two photon ionization (R2PI), IR-UV, and UV-UV double resonance spectroscopic techniques, and quantum chemical calculations. In their experiments N–H…π hydrogen bonds, slanted T-shaped structures, were observed in molecular dimer. The experimental observations could be the evidence of Hp-π bonds in molecular interactions.

The polar hydrogen-π (Hp-π) interactions are very different from the non polar hydrogen-π (H-π or CH-π) interactions in interaction strength, and in physical nature and properties. The main physical contributions in Hp-π interactions are the electrostatic interactions, MO coordinating, and electron dispersion interaction. The Hp-π interactions are distance and orientation dependent, and the best orientation is the perpendicular direction from polar hydrogen atom to the π-plane. The interaction energies of polar hydrogen-π interactions (Hp-π) are much stronger than that of the disputed non polar hydrogen-π interactions (CH-π) [8,9].

The three dimensional structures of proteins are not rigid constructions, but dynamically flexible. The Hp-π interactions are point to π-plane interactions, possessing much more interaction conformations and broader energy range than that of the common hydrogen bond interactions. The Hp-π interactions may be responsible for the flexibility and dynamic activity of proteins. In biological molecules the polar hydrogen atoms are the common interaction donors of both hydrogen bond interactions and Hp-π interactions. These two interaction types happen at different directions. The most favorable orientation (perpendicular direction) for the Hp-π interactions is just the most unfavorable direction for common hydrogen bond interactions. Consequently we can expect that the common hydrogen bond (H-b) interactions and the polar hydrogen-π (Hp-π) interactions are the two main interaction forces supporting the three dimensional structures of proteins, and playing their roles in different directions.

Based on our calculations, the Hp-π energies in solutions are decrease with the increase of the solvent dielectric constants ϵ. In this study the Hp-π interaction energies in solutions are calculated using PCM (Polarized Continuum Model) method. The PCM is a continuing medium model, which cannot give very accurate results. The Hp-π energies in aqueous solution are still significant. At this point the Hp-π interactions are like common hydrogen bond interaction, lees affected by solvation effects. The Hp-π interaction acceptors (aromatic groups) are often the hydrophilic groups, which are buried in the hydrophobic core of protein structures. In the hydrophobic pockets of proteins the solvent dielectric constants are small, and the Hp-π interactions may be still working well.

## Conclusion

The polar hydrogen-π interactions (Hp-π) are a unique interaction type different from other main molecular interaction types in physical nature and properties. Some useful conclusions can be refined as follows. (1) In the 20 natural amino acids 11 of them are involved in the Hp-π interactions, including Ser, Thr, Gln, Asn, Arg, Lys, Phe, Tyr, Trp, Cys, and His. In proteins the Hp-π interaction donors are the atomic groups –NH_2_, >NH, –OH, –SH, and C_6_H_5_OH; while the Hp-π interaction acceptors are various aromatic and heteroatom aromatic groups in amino acid side chains. (2) The peptide bond units in protein backbones are quasi π-bonds, possessing both polar hydrogen atoms and conjugate π-groups, playing the roles of both Hp-π interaction acceptors and donors. (3) The Hp-π interactions are point to π-plane interactions, having many possible interaction conformations. (4) The Hp-π interaction energies between amino acids are in the range from -10 to -25 kJ/mol, close or even larger than the common hydrogen bonds. (5) The bond length of Hp-π interactions are in the region from 2.30 to 3.00 Å at the perpendicular direction to the π-plane, much longer than common hydrogen bonds. (6) Like common hydrogen bond interactions, the Hp-π interactions are less affected by solvation effects. (7) The common DFT method B3LYP fails in describing the Hp-π interactions. On the other hand, CCSD/6-311 + G(d,p) plus ghost atom H-Bq at bond middle can yield better results using less cpu-time, very close to the state-of-the-art method CCSD(T)/cc-pVTZ.

## Method and theory

DFT method B3LYP has been widely used in the studies of organic molecules and biological molecules for many years because of its higher accuracy and less calculation workload. However, in recent decade the common DFT methods were found failing in description of molecular dispersion interactions, which are a main contribution in Hp-π interactions. On the other hand, more advanced quantum chemical configuration interaction (CI) [39-41] methods are able to evaluate the dispersion energies. However, such sophisticated methods are expensive, consuming much more CPU times and computer resource than DFT methods do. Particularly the typical bond lengths of Hp-π interactions are around 2.5 Å, much longer than other molecular interaction types, such as hydrogen bonds (~2.0 Å). In order to calculate the long range Hp-π interactions accurately large basis sets have to be used. The large basis sets make the CI calculations of Hp-π interactions are even more expensive and CUP-time consuming. Careful selection of appropriable methods for the Hp-π interaction calculations is the first step of the Hp-π study.

A comprehensive comparison was performed to evaluate the calculation results of several methods, including three methods (B3LYP, CCSD, and CCSD(T)) [42-45] and three basis sets (6-311 + G(d,p), TZVP, and cc-pVTZ) [46,47]. Small Hp-π interaction donors (CH_3_OH) and two acceptors (C_2_H_4_ and C_6_H_6_) are used in the comparison calculations. The Hp-π interaction energy of CH_3_OH-C_2_H_4_, one of the smallest Hp-π interaction pairs, is -11.853 kJ/mol, more than half of the H_2_O-H_2_O hydrogen bond energy -21.258 kJ/mol. The Hp-π interaction energy of CH_3_OH-C_6_H_6_ is -19.765 kJ/mol, very close to the common hydrogen bonds. In biological molecular interactions the Hp-π interactions are comparable to the hydrogen bond interactions. A remarkable difference between Hp-π interactions and H-b interactions is that the hydrogen bonds are point to point interactions, and the Hp-π bonds are point to π-plane interactions.

The Hp-π interaction structures and HOMO (highest occupied molecular orbital) of the Hp-π interaction pairs are shows in Figure 1. In the interaction pair CH_3_OH-C_2_H_4_ the polar hydrogen of CH_3_OH points to the double bond center of C_2_H_4_ perpendicularly, and the polar hydrogen atom points to the carbon and the bond center of C_2_H_4_, respectively. In the HOMO figures of CH_3_OH-C_2_H_4_ interaction pair the polar hydrogen atoms are in close touching with the π-orbitals of C_2_H_4_. At this point Hp-π interactions are similar to the hydrogen bond interactions. In the latter the polar hydrogen atoms are buried in the electron density of electronegative atoms, such as oxygen and nitrogen.

The more advanced CI method CCSD (T) gives better results than that of the CCSD method. However, the cpu-time of CCSD (T) is much longer than that of the CCSD. Generally speaking the larger basis sets give the better results. In the calculations of Hp-π interactions polarization functions, diffuse functions, and floating functions are necessary. However, large basis set remarkably increases the cpu-time of CCSD (T) calculations. In solving this problem a simple method is the use of ‘ghost atoms’. The ghost hydrogen atom H-Bq is an empty atom possessing the basis functions of hydrogen, but having no nucleus charge and electron [48,49]. In the CCSD calculation using 6-311 + G(d,p) basis set plus a hydrogen ‘ghost atom’ H-Bq yields the result (-11.715 kJ/mol), very close to the result (-11.853 kJ/mol) of the state-of-the-art method CCSD(T)/cc-pVTZ. However, the cpu-time of CCSD calculation reduces to 1/8 of CCSD(T) calculation (5.3 hours to 44.6 hours). Another advantage of the use of ghost atom is reducing the basis set superposition error (BSSE) [48,49].

In the Hp-π interaction calculations by using DFT method B3LYP only take cpu-time few minutes. However, the Hp-π interaction energies are around 20 ~ 30% smaller than that of other two CI methods (CCSD and CCSD (T)), because the common DFT methods fail in evaluating the dispersion energies, which is an important contribution in Hp-π interactions. In recent years the shortcoming of DFT methods has been improved by the better density functionals or using empirical correction for dispersion.

In this study all calculations are performed using CCSD method and basis set 6-311 + G(d,p) + H-Bq, in which a ‘ghost hydrogen atom’ (H-Bq) is attached to the polar hydrogen atom, and the distance to polar hydrogen atom is 0.9 Å. Keep in mind, in the calculations for Hp-π interaction energies the same ghost atom is also added to the two molecule monomers. The Hp-π interaction energies in solutions are calculated using CCSD and PCM (Polarized Continuum Model) method [34-37]. All calculations are performed at Sugon-5000A computer and Tianhe-1A computer in National Supper Computing Center in Tianjin (China) using Gaussian 09 and Gauss View 5 software packages [50].

## Competing interests

All authors declare that there are no any competing interests.

## Authors’ contributions

All authors contributed equality for the development of the manuscript. RBH and QSD designed the research scheme and wrote the article. QYW did most calculations, LQD and DC performed the data collection and analysis. All authors read and approved the final manuscript.
